# LIF/LIFR Signaling in Gastric Cancer: A Double‐Edged Sword in Tumor Progression and Therapeutic Resistance

**DOI:** 10.1002/cam4.71315

**Published:** 2025-10-29

**Authors:** Daniel Park, Kyung‐Il Kim, Yate‐Ching Yuan, Pranati Shah, Hannah Zhong, Yu‐Han Chen, Sharity Ondrejik, Ellen Choi, Sofia Guzman, Vitor Goes, Christiana Crook, Wenqi Wang, Dani Ran Castillo, Daneng Li

**Affiliations:** ^1^ Department of Internal Medicine University of California San Francisco – Fresno Campus Fresno California USA; ^2^ Department of Internal Medicine Kaiser Permanente Inland Empire Fontana California USA; ^3^ Translational Bioinformatics Center for Informatics City of Hope Duarte California USA; ^4^ Department of Internal Medicine Loma Linda University Loma Linda California USA; ^5^ Department of Hematology and Hematopoietic Cell Transplantation City of Hope Duarte California USA; ^6^ Department of Internal Medicine Englewood Hospital and Medical Center Englewood New Jersey USA; ^7^ Department of Medical Oncology & Therapeutics Research City of Hope Comprehensive Cancer Center Duarte California USA; ^8^ Department of Internal Medicine Hospital Israelita Albert Einstein São Paulo São Paulo Brazil; ^9^ Department of Developmental and Cell Biology University of California Irvine California USA

**Keywords:** biomarker, gastric cancer, leukemia inhibitory factor, leukemia inhibitory factor receptor, pleiotropy, signaling pathways

## Abstract

Gastric cancer (GC) remains a major cause of cancer‐related mortality worldwide, driven by late‐stage diagnoses and poor survival outcomes. Leukemia inhibitory factor (LIF) and leukemia inhibitory factor receptor (LIFR) are increasingly recognized as critical players in GC pathophysiology, though their exact roles are not fully understood. LIF, a multifunctional cytokine in the interleukin‐6 family, signals through the LIFR/gp130 complex and activates oncogenic pathways such as JAK/STAT3, MAPK/ERK, and Hippo‐YAP. Emerging evidence indicates that LIF drives tumor progression by promoting epithelial–mesenchymal transition, immune evasion, and chemoresistance. Clinically, high LIF expression is associated with poor prognosis, peritoneal metastasis, and resistance to chemotherapy and immunotherapy. This review explores the molecular mechanisms of LIF/LIFR signaling in GC, highlighting its potential as a prognostic biomarker and therapeutic target.

**Mini Abstract Conclusion:**

The LIF/LIFR pathway contributing to gastric cancer is complex with context‐dependent pro‐ versus anti‐tumorigenic roles in GC progression, marking it as a significant biomarker and target for future therapies.

## 1 Introduction

Gastric cancer (GC) is the fourth‐leading cause of cancer‐related deaths worldwide [1]. According to the World Health Organization's International Agency for Research on Cancer (IARC), there were approximately 970,000 new cases and 660,000 deaths due to GC in 2022. The highest incidence of GC has been observed in East Asia, accounting for 71.4% of new GC cases and 70.1% of GC‐related deaths globally. Gastric cancer (GC) continues to pose a major global health challenge. According to the most recent GLOBOCAN estimates, there were over 1.1 million new GC cases and 770,000 deaths worldwide in 2020, ranking it as the fifth most common cancer and the fourth leading cause of cancer‐related mortality globally. Despite ongoing advances in systemic therapy, the 5‐year survival rate for patients with advanced or metastatic disease remains dismal, approximately 7% in the United States and similarly low across non‐Asian populations. One of the greatest barriers to progress is the limited set of clinically actionable biomarkers, with current therapeutic decisions largely guided by HER2, PD‐L1, and MSI status. However, tumor heterogeneity and variability in biomarker expression hinder consistent therapeutic benefit [2, 3].

Emerging targets such as Claudin 18.2 (CLDN18.2) and fibroblast growth factor receptor 2b (FGFR2b), now detectable via immunohistochemistry (IHC), are gaining clinical traction, with CLDN18.2‐targeted therapies like zolbetuximab showing promise in recent trials [4]. These developments reflect a broader shift toward precision oncology in gastric cancer, underscoring the need for continued biomarker discovery and validation. In this context, the LIF/LIFR signaling axis represents a compelling and understudied pathway involved in tumor growth, immune evasion, and therapeutic resistance [5]. Comprehensive profiling of LIF/LIFR activity and its downstream networks, such as Hippo/YAP and STAT3, may not only help identify patient subsets with elevated signaling activity but also inform the development of novel therapeutic combinations with immune checkpoint inhibitors or TAM reprogramming agents. Expanding the biomarker landscape to include LIF/LIFR, CLDN18.2, FGFR2b, and other emerging targets is critical to advancing personalized treatment strategies and improving survival outcomes for patients with advanced gastric cancer.

In efforts to improve long‐term outcomes, cytokines and molecular markers involved in tumorigenesis have been explored. Leukemia inhibitory factor (LIF) and its receptor, leukemia inhibitory factor receptor (LIFR), have been identified as being overexpressed in GC tissues; their role in tumor differentiation, lymphovascular invasion, and metastasis is under investigation [6].

LIF is a multifunctional cytokine and member of the interleukin (IL)‐6 family. It exerts pleiotropic effects that are highly dependent on cell type and organ context. LIF activates signaling pathways by binding to the LIFR/glycoprotein 130 (LIFR/gp130) heterodimer, which initiates signal transduction through pathways such as the Janus kinase (JAK) and Src‐homology‐2 domain–containing signal transducer and activator of transcription 3 (STAT3) pathway [7, 8]. Beyond JAK/STAT3 activation, LIF has been implicated in pro‐tumorigenic processes through the LIFR/Hippo/Yes‐associated protein (YAP) pathway, further emphasizing its role in cancer biology [9].

To date, elevated expression of LIF has been associated with poor prognosis and distant metastasis in breast cancer, malignant melanoma, colorectal cancer, and pancreatic cancer [9, 10, 11, 12]. However, the specific role of the LIF/LIFR axis in GC remains incompletely understood. This review aims to provide a comprehensive analysis of the current understanding of LIF and LIFR in the context of GC, highlighting molecular mechanisms of action, contributions to tumor progression, and potential as therapeutic targets.

## 2 LIF/LIFR Signaling Pathways

LIF signaling is mediated by LIFR/gp130 heterodimerization [13, 14]. Gp130 is a shared component among other members of the IL‐6 cytokine family. In the traditional LIF/LIFR pathway, the interaction between LIF and LIFR activates JAK, leading to the phosphorylation of STAT3. Phosphorylated STAT3 forms homodimers, translocates to the nucleus, and drives the expression of target genes such as c‐MYC, MMP2, and c‐FOS [13, 15].

The binding of LIF to LIFR can activate additional signaling cascades. Members of the JAK family, including JAK1, JAK2, and TYK2, can associate with LIFR; however, JAK1 is believed to play a dominant role, as LIF/LIFR signaling is significantly impaired in its absence. Upon LIF binding, JAK1 phosphorylates specific tyrosine residues on gp130 and gp190, which act as scaffold proteins to facilitate downstream signaling processes. For example, phosphorylation of gp130 residues Y767, Y814, and Y915, and gp190 residues Y98 and Y1028 facilitate STAT3 binding. In contrast, phosphorylation of Y759 on gp130 and Y974 on gp190 enables the recruitment of GRB2 through its SHP2 domain, activating the MAPK signaling pathway [16].

LIF/LIFR signaling activates the MAPK/ERK cascade through GRB2 phosphorylation, which subsequently leads to the activation of RAF, RAS, MEK, and ERK [17, 18]. A key regulatory mechanism of this pathway involves suppressor of cytokine signaling 3 (SOCS3), a feedback inhibitor induced by STAT3, which directly binds to JAK, inhibiting the initiation of intracellular signaling. SOCS3 and GRB2 share the same binding sites on gp130 and gp190, as both interact via their SHP2 domain [19]. This competitive binding mechanism enables SOCS3 to suppress LIF‐mediated MAPK activation.

**FIGURE 1:**
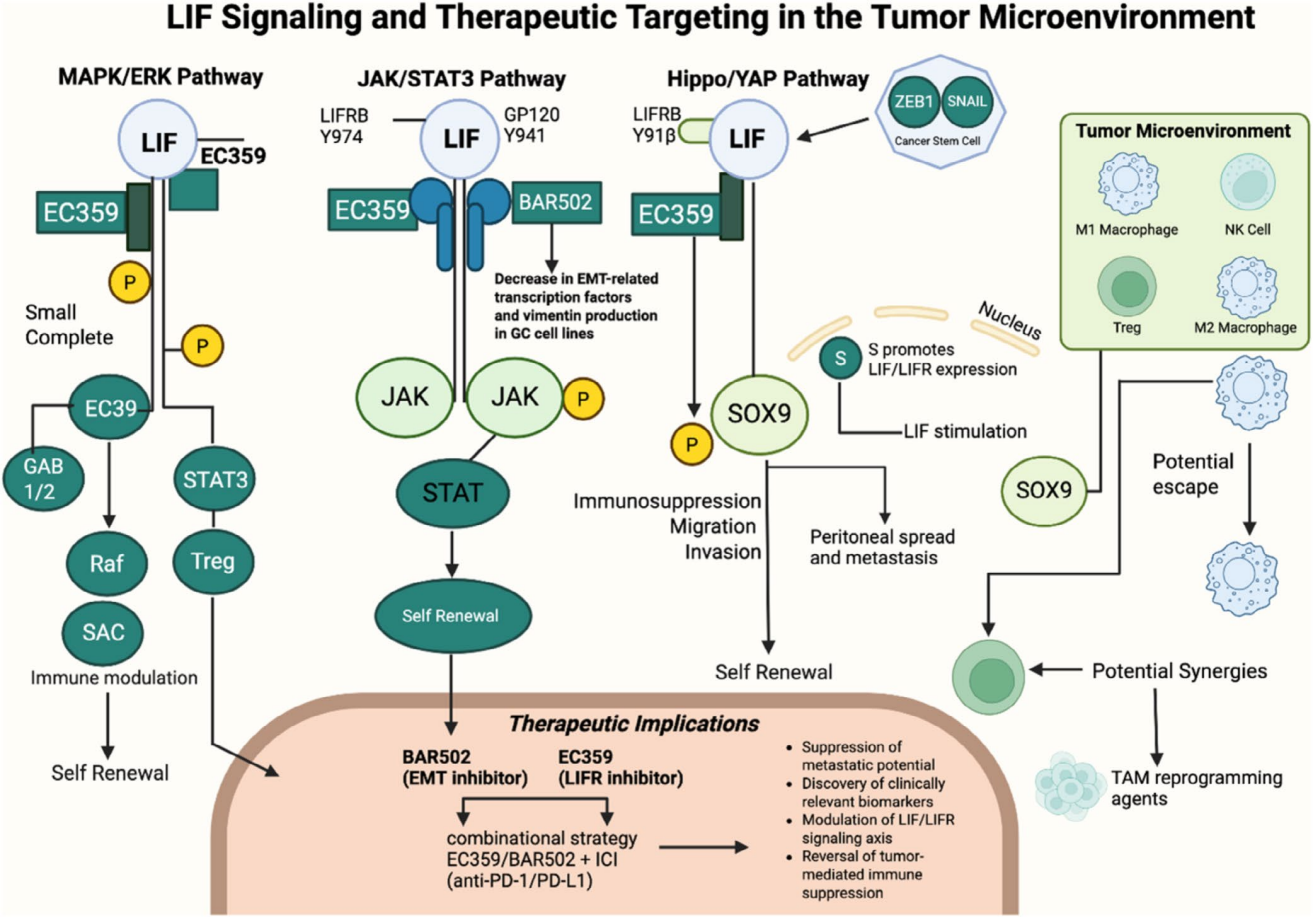
Signal transduction pathways of LIF/LIFR including MAPK/ERK, JAK/STAT, and Hippo Pathway. ERK, extracellular signal‐regulated kinase; JAK, Janus kinase; LIF, leukemia inhibitor factor; LIFR, leukemia inhibitory factor receptor; MAPK, mitogen‐activated protein kinase; SOX9: SRY (sex‐determining region Y)‐box 9; STAT, signal transducer and activator of transcription.

Recent literature has demonstrated a potential LIFR/Hippo/YAP pathway in tumorigenesis [9]. The Hippo pathway plays a critical role in cancer progression, stem cell renewal, and organ regeneration [9, 20]. YAP is a downstream transcriptional coactivator in the Hippo pathway. When Hippo signaling is disrupted, YAP translocates into the nucleus, activating multiple oncogenes [21]. Notably, YAP/TAZ is frequently upregulated in solid tumors and drives tumor growth, while the loss of Hippo signaling contributes to cancer development by enhancing proliferation and inhibiting apoptosis [22]. In GC, YAP nuclear translocation has been linked to worse prognosis via LIF stimulation, which has been shown to inactivate Hippo signaling by reducing phosphorylation of MST1, LATS1, and YAP, ultimately facilitating YAP nuclear translocation and promoting GC cell proliferation and migration [9]. Additionally, LIF and LIFR expression levels are higher in GC cells compared to normal tissue and have been linked with tumor differentiation, increased lymph node metastasis, and higher rates of lymphovascular invasion [13]. Additional studies suggest that LIF/LIFR engagement influences multiple downstream pathways, including Rap1 and PI3K‐AKT, indicating a context‐dependent role of LIF/LIFR in different cellular processes [13]. Figure 1 details signal transduction pathways of LIF/LIFR.

## 3 Tumor‐Promoting Roles of LIF/LIFR in GC

Bian et al. demonstrated that LIF and LIFR are overexpressed in GC tissues and significantly associated with aggressive tumor features, including poor differentiation, lymphovascular invasion, advanced tumor stage, lymph node metastasis, and higher pathological TNM stage [9]. Functionally, LIF promoted GC cell proliferation, colony formation, invasion, migration, and in vivo tumor growth. It also enhanced cell cycle progression and inhibited apoptosis [9, 23].

These effects were largely reversed by LIFR knockout, highlighting a key role for LIFR in mediating LIF‐driven oncogenic processes [24]. To further characterize these mechanisms, in vitro studies were conducted to assess the impact of LIF on cell cycle and apoptosis. In shRNA‐transfected GC cells, LIF treatment significantly reduced the proportion of cells in the G1 phase, indicating cell cycle progression. Interestingly, the knockdown of LIFR did not fully reverse this effect, suggesting that LIF may also promote cell cycle progression via LIFR‐independent pathways. In contrast, LIF's antiapoptotic effects were found to be LIFR‐dependent: LIF reduced the proportion of early apoptotic cells and increased viable cell numbers, and these effects were attenuated when LIFR was silenced. The study also investigated whether LIF regulates GC cell proliferation by modulating the Hippo–YAP signaling pathway through LIFR. At the protein level, it revealed that LIF suppressed the phosphorylation of MST1, LATS1, and YAP in a dose‐dependent manner, while total YAP levels in both the cytoplasm and nucleus remained largely unchanged. The ratio of phosphorylated YAP to total YAP (p‐YAP/YAP) decreased with increasing LIF concentrations, indicating suppression of Hippo pathway activity. Importantly, LIFR knockdown attenuated these effects. Although total Scribble protein expression remained unchanged, its localization to the plasma membrane was reduced by LIF and restored upon LIFR knockdown. These findings suggest that LIF regulates GC cell proliferation through LIFR‐mediated suppression of the Hippo–YAP signaling pathway [9].

Another study tried to explore the role of LIF/LIFR signaling in GC using MKN45 cell line and demonstrated that LIF promotes cell proliferation, cell cycle progression, and EMT in a concentration‐dependent manner. Notably, higher concentrations of LIF led to growth retardation, suggesting potential cytotoxic effects. In contrast, at lower concentrations, LIF reduced the percentage of cells in the G0–G1 phase while increasing those in the S–G2–M phases, indicating accelerated cell cycle progression. LIF also downregulated E‐cadherin and upregulated vimentin and SNAIL1, supporting its role in EMT induction; however, these effects diminished at cytotoxic concentrations. To assess the therapeutic potential of targeting LIFR, the selective LIFR inhibitor EC359 (a small molecule inhibitor that selectively binds LIFR and downregulates its pro‐oncogenic effects in vitro and in vivo, causing a disruption in the LIF‐mediated activation of STAT3, mTOR, and AKT pathways [25, 26]) was applied, which effectively reversed LIF‐induced proliferation and EMT markers in a dose‐dependent manner. EC359 significantly suppressed cMYC expression and inhibited cell cycle progression, with the effects becoming evident at 25 nM. While EC359 alone did not impair basal proliferation, it attenuated LIF‐driven oncogenic activity, suggesting that LIFR blockade may be a viable strategy to counteract LIF‐mediated tumor progression in GC [9, 27].

GC with peritoneal carcinomatosis (PC) is associated with particularly poor prognosis, as these patients often show limited response to both chemotherapy and immunotherapy. A recent study focusing on GC stem cells revealed that SOX9 plays a key role in regulating immune‐related pathways in GC with PC. RNA‐seq analysis of SOX9 knockout (KO) versus control GA0518 cells identified significant differential expression of 71 genes, including the downregulation of secreted factors such as LIF and CX3CL1. Gene Set Enrichment Analysis (GSEA) showed that 15 hallmark pathways were significantly altered upon SOX9 KO, including five immune‐related pathways such as interferon‐γ and α responses, TNF signaling via NF‐κB, allograft rejection, and general inflammatory responses. Among the most downregulated cytokines was LIF. High LIF expression correlated with shorter survival in GC patients. Bulk RNA‐seq and single‐cell RNA‐seq of 37 PC specimens revealed enrichment and positive correlation of SOX9, LIF, LIFR, and IL6ST (GP130) in tumor cells. SOX9 KO in both GA0518 and AGS cells led to significantly decreased LIF transcription and secretion; LIF and SOX9 were co‐expressed in tumor cells, and LIF was elevated in malignant ascites but undetectable in plasma and cytology‐negative peritoneal washings. Functionally, recombinant LIF activated phospho‐STAT3 and phospho‐S6 in GA0518 cells, while LIF‐neutralizing antibody or the LIF/LIFR inhibitor EC359 reduced STAT3 phosphorylation and LIF‐induced invasiveness. Collectively, these findings demonstrate that LIF is a critical mediator of tumor progression and immunosuppression in peritoneal carcinomatosis and may serve as a therapeutic target and prognostic biomarker in this aggressive GC subset [28].

## 4 LIF/LIFR and Its Impact on the Gastric Cancer Tumor Microenvironment

The role of LIF/LIFR signaling in the tumor microenvironment (TME) is a topic of growing interest, particularly its interactions with immune cells and transcriptional regulators [29, 30]. Tumor‐associated macrophages (TAMs) are the most abundant immune cells within the TME of solid tumors, playing a pivotal role in driving tumor progression and chemoresistance [31, 32]. While M1 TAMs are known for their anti‐tumor functions and M2 TAMs are known for their tumor‐supporting, anti‐inflammatory roles, the precise mechanisms by which M2 TAMs promote chemoresistance remain unclear [33, 34]. Notably, Yu et al. demonstrated that LIF directly induces M2 polarization of macrophages through activation of the LIFR/STAT3 pathway in GC [35]. The cytotoxic chemotherapy drug, cisplatin, was shown to increase the presence and activation of M2 TAMs, while tumors overexpressing LIF exhibited significantly higher levels of M2‐associated marker genes. Furthermore, LIF directly promoted the activation of M2‐type macrophages in both bone marrow–derived and THP‐1 cell–derived macrophages. This effect was shown to be STAT3‐dependent, as STAT3 deletion attenuated LIF‐induced M2 polarization and reduced M2‐type TAMs in cisplatin‐treated GC tumors [35].

Additionally, Yu et al. demonstrated that chemotherapy reduces LIF expression in GC cells through the activation of HIF1α signaling [35]. In their findings, cisplatin and oxaliplatin induced the accumulation of HIF1α, which is essential for the upregulation of LIF transcription. Silencing HIF1α significantly reduced chemotherapy‐induced LIF mRNA levels, highlighting its critical role in this pathway. Further analysis revealed that HIF1α directly binds to a hypoxia‐responsive element (HRE2) in the LIF promoter region, as the deletion of HRE2 abolished chemotherapy‐induced LIF promoter activity. Chromatin immunoprecipitation assays confirmed this interaction, demonstrating that chemotherapy or overexpression of a constitutively active HIF1α mutant enhanced HIF1α binding to the HRE2 region [35].

Recent studies have further investigated the downstream oncogenic effects of LIF/LIFR signaling, particularly through its regulation of FGFR4 [36, 37]. LIF was shown to upregulate FGFR4 expression in GC via STAT3 activation, with higher FGFR4 levels correlating with worse outcomes in diffuse‐type GC [37]. Functional studies in MKN45 cells and patient‐derived organoids demonstrated that LIF exposure significantly increased FGFR4 expression and STAT3 activation, promoting a hyperproliferative and chemoresistant phenotype. Targeting this axis with the LIFR antagonist LRI‐201 reversed these effects by inhibiting LIFR‐LIF interactions and reducing FGFR4 expression. LRI‐201 also curtailed tumor cell migration, which limited wound closure rates in scratch assays and increased apoptosis, as indicated by Annexin V staining. Furthermore, the findings highlighted a reciprocal regulation between the LIF/LIFR/STAT3 and FGF19/FGFR4 pathways, with FGFR4 inhibition via roblitinib essentially reversing the pro‐oncogenic properties of LIF [37]. These results emphasize the intertwined roles of LIF pathways in driving epithelial–mesenchymal transition.

Although not yet fully explored in GC, LIF has also been shown to play a key role in regulating immune cell infiltration within the TME. Cancer response to immunotherapy is highly dependent on CD8+ T cell infiltration and the presence of TAMs, yet the factors governing these processes remain poorly understood [38, 39]. Pascual Garcia et al. found that LIF actively suppresses CD8+ T cell recruitment while promoting an immunosuppressive environment through TAMs [38]. Blocking LIF in tumors with high LIF expression decreased the levels of pro‐tumoral TAM markers such as CD206, CD163, and CCL2 and induced CXCL9 expression, which facilitates CD8+ T cell infiltration. Mechanistically, LIF suppresses CXCL9 transcription through epigenetic silencing, preventing it from acting as a chemoattractant for cytotoxic T cells. Importantly, combining LIF inhibition with PD‐1 checkpoint blockade in murine models demonstrated enhanced tumor regression [38].

## 5 Clinical Implications of LIF/LIFR in Gastric Cancer: Impact on Tumor Aggressiveness and Metastatic Potential

Despite various preclinical studies on LIF/LIFR, limited clinical data exist. Sabry et al. investigated the relationship between LIF and IL‐11 expression in *H. pylori* ‐infected patients with gastritis or GC, revealing that both cytokines were significantly elevated in patients with GC compared to those with gastritis [40]. Median LIF expression was significantly higher at 1.68 pg/mL in GC patients versus 0.55 pg/mL in gastritis patients, while IL‐11 expression was 0.63 pg/mL in GC patients compared to 0.14 pg/mL in gastritis patients. The findings demonstrated a strong correlation between *H. pylori* bacterial load, LIF expression, and IL‐11 expression, suggesting that persistent *H. pylori* infection drives an inflammatory cascade that may contribute to tumor progression. Notably, the highest LIF expression was observed in high‐grade adenocarcinomas in grade III and correlated with increased tumor severity [40].

Transcriptomic analyses have further demonstrated the role of the LIF/LIFR axis in GC progression. Di Giorgio et al. conducted high‐throughput sequencing on paired gastric tissue samples from 31 patients with and without peritoneal carcinomatosis (PC); they identified LIFR as one of the most significantly upregulated genes in patients with PC and a predictor of poor prognosis [27]. Patients with peritoneal involvement had a median survival of 14.5 months, significantly shorter than the 53‐month median survival in those without peritoneal disease (5‐year survival rates: 25% vs. 49.2%). In vitro studies using MKN45 GC cells further demonstrated that LIF stimulation led to a concentration‐dependent increase in proliferation and epithelial–mesenchymal transition, marked by E‐cadherin suppression and vimentin upregulation. These effects were driven by JAK‐STAT3 phosphorylation and acquisition of a migratory phenotype, which facilitated adhesion to the peritoneum in an ex vivo model. Importantly, pharmacologic inhibition of LIFR using EC359 reversed these oncogenic effects, attenuating cell proliferation, EMT markers, and migration. In vitro, EC359 significantly counteracted the effects of LIF by cell cycle arrest and apoptosis, blocking the LIF‐induced transition from the resting (G0–G1) phase to the proliferative (S–G2–M) phase and increasing apoptosis rates that LIF had reduced. In MKN45 cells treated with LIF, EC359 reversed EMT features by modulating marker expression, it downregulated E‐cadherin and reduced vimentin levels [27]. Overall, these findings emphasize the LIF/LIFR axis as a key driver of peritoneal metastasis in GC and reinforce its potential as a therapeutic target. Building on the repositioning of mifepristone as a LIFR antagonist, Christina Di Giorge et al. developed a novel small‐molecule LIFR inhibitor, LRI‐201 and evaluated its activity through both in vitro and in silico models [37]. Molecular dynamics simulations demonstrated that, despite the high flexibility of the surrounding loops, LRI‐201 maintains a stable binding conformation within the LIFR ligand‐binding pocket formed by loops L2 and L3. Key interactions include a hydrogen bond between the carbonyl group at position 3 and Thr308 (L2 loop), and hydrophobic interactions between the allyl double bond and residues Tyr318, Leu331, Tyr342, Leu359, and Trp302 (L3 loop). The biphenyl moiety of LRI‐201 extends toward the LIF‐binding interface, engaging Pro337 via hydrophobic contact. This binding mode significantly alters the LIF‐binding pocket architecture, increasing the distance between L2 and L3 compared to the native hLIF–mLIFR structure. Functionally, LRI‐201 inhibited LIF/LIFR interaction in an AlphaScreen assay with an IC₅₀ of 21.92 ± 2.16 μM and effectively reversed LIF‐induced proliferation, epithelial–mesenchymal transition (EMT), and capecitabine resistance in MKN45 GC cells [37].

The effects of BAR502, a non‐bile steroidal ligand of the Farnesoid X Receptor (FXR) and G Protein–Coupled Bile Acid Receptor 1 (GPBAR1), have been investigated on tumor proliferation and epithelial‐mesenchymal transition (EMT), with a focus on selective LIFR antagonism. In MTS assays, BAR502 treatment significantly inhibited the proliferation of pancreatic ductal adenocarcinoma (PDAC) cell lines in a concentration‐dependent manner. The most pronounced antiproliferative effect was observed in cells treated with BAR502 (20 μM) plus LIF, which showed a proliferation index of < 1—significantly lower than both the untreated (NT) group and the LIF‐only group. BAR502 also suppressed EMT, evidenced by a significant reduction in vimentin mRNA expression compared to LIF‐treated cells, and by wound healing assays, which showed ~40% closure in the BAR502 group versus 16% in the LIF group. While this study was conducted in PDAC cells, the downstream effects of BAR502—particularly LIFR antagonism and vimentin downregulation—are also relevant in gastric cancer (GC). Future studies are warranted to evaluate the effects of BAR502 in GC cell lines and assess its potential clinical utility in this context [41].

In addition to its role in tumor progression, LIF has been associated with immune‐related toxicity in patients with gastrointestinal (GI) cancers receiving immune checkpoint inhibitors [42, 43]. In a retrospective study of 51 patients with metastatic GI cancer, 17 of whom had GC, elevated LIF levels correlated with myositis; affected patients exhibited a median LIF concentration of 21.39 pg/mL compared to 12.72 pg/mL in those without myositis. Given LIF's established involvement in inflammation and immune regulation, these findings suggest its potential as both a driver of tumor progression and a predictive biomarker for immune‐related adverse events in ICI‐treated patients. Table 1 provides an overview of the literature on LIF/LIFR in GC.

**TABLE 1:** Summary of studies investigating LIF/LIFR in gastric cancer.

Author	Country	Study type	Subjects	Sample size	Methodology	Key findings	Conclusion
Sabry et al. (2018) [40]	Egypt	Case–control	*H. pylori* ‐infected Egyptian patients with gastritis and GC	147	Gastric lesion biopsies and qRT‐PCR to detect *H. pylori* load, LIF, and IL‐11 expression	*H. pylori* load, LIF, and IL‐11 were significantly higher in GC patients than in gastritis patients.	LIF is a potential immunotherapeutic target for *H. pylori* ‐associated GC.
Xu et al. (2019) [44]	China	Case–control	Patients with primary GC	80	Analysis of GC tissues and matched adjacent non‐neoplastic tissues (ANT)	GC tissues had lower LIF expression than ANT. The 5‐year survival rate was significantly lower in GC patients with LIF downregulation.	LIF expression is an independent prognostic factor for survival in GC patients.
Di Giorgio et al. (2022) [27]	Italy	Case series	Patients with GC undergoing surgery	31	RT‐PCR analysis of LIFR expression in GC tissues compared to non‐neoplastic mucosa; comparison of GC patients with and without peritoneal disease	LIFR expression was higher in patients with peritoneal involvement.	LIFR is a strong predictor of poor prognosis and a negative prognostic factor for GC with carcinomatosis.
Wang et al. (2022) [42]	China	Cohort	Metastatic GI cancer patients	51	Serum biomarker panel testing for immune‐related adverse events (irAEs) and prognosis correlation	Increased IL‐1a, IL‐21, LIF, and PIGF‐1 levels were linked to myositis incidence/irAEs. Patients with irAEs had better tumor prognosis.	Serological proteins are promising markers for predicting immune‐related toxicity and prognosis in GI cancer patients.
Seeneevassen et al. (2020) [45]	France	Cohort	GC patients	177	Analysis of paraffin‐embedded GC samples and survival probability based on ZEB1 and LIFR expression	High LIFR expression was associated with better clinical outcomes in GC patients.	LIFR is a novel prognostic marker for GC.
Zhang et al. (2023) [46]	China	Cohort	RNA sequences from GC and non‐tumor samples (TCGA database)	375	Bioinformatics analysis using R ‘limma’ package to identify metastasis‐related differentially expressed genes	LIF gene expression correlated with immune cells (e.g., neutrophils), age, and tumor grade.	LIF could serve as a prognostic marker and potential therapeutic target.
Di Giorgio et al. (2024) [37]	Italy	Cohort	Paired GC samples (neoplastic and non‐neoplastic tissues)	31	RNA analysis of paired GC samples collected during surgery	LIFR expression correlated directly with FGFR4 in GC tissues; FGFR4 is a downstream target of the LIF/LIFR complex.	LIF and FGF19 regulate oncogenic STAT3 signaling in GC cells.

Abbreviations: ANT, adjacent non‐neoplastic tissues; FGF19, fibroblast growth factor 19; FGFR4, fibroblast growth factor receptor 4; GC, gastric cancer; GI, gastrointestinal; *H. pylori* , *Helicobacter pylori* ; IL‐11, interleukin‐11; IL‐1a, interleukin‐1 alpha; IL‐21, interleukin‐21; irAEs, immune‐related adverse events; LIF, leukemia inhibitory factor; LIFR, leukemia inhibitory factor receptor; PIGF‐1, placental growth factor‐1; qRT‐PCR, quantitative reverse transcription polymerase chain reaction; STAT3, signal transducer and activator of transcription 3; TCGA, The Cancer Genome Atlas; ZEB1, zinc finger E‐box binding homeobox 1.

## 6 Potential Anti‐Tumorigenicity of LIF/LIFR in Gastric Cancer

Although preclinical and clinical studies have established pro‐tumorigenic properties of LIF and LIFR by facilitating M2 macrophage polarization, growth factor upregulation, and inflammatory cytokine release, emerging research suggests that LIF and LIFR may also exhibit anti‐tumorigenic effects under certain conditions [7, 47].

Given the increasing relevance of microRNAs as cancer biomarkers, Zhang et al. explored the role of miR‐589 in GC progression through its interaction with LIFR and the PI3K/AKT/c‐Jun pathway, a key driver of cellular proliferation [48]. This study showed that miR‐589 directly suppresses LIFR expression, thereby enhancing PI3K/AKT/c‐Jun signaling and acting as a tumor‐promoting factor in GC tumor specimens. Quantitative RT‐PCR and Western blot analyses revealed a four‐fold decrease in LIFR expression in miR‐589–treated GC cells compared to controls. Furthermore, miR‐589–transfected cells treated with LIFR exhibited a two‐fold reduction in cellular invasion, supporting the role of LIFR as a potential tumor suppressor.

Xu et al. further investigated the anti‐tumor potential of LIF by examining its effect on cell cycle regulation in GC [44]. Flow cytometry analysis of LIF‐overexpressing GC cells demonstrated a significant increase in G1 phase cell cycle arrest compared to controls. Western blot analysis revealed that LIF suppressed the expression of cyclin D1, a key cell cycle promoter, and increased the expression of p21, a known cell cycle inhibitor. Although the precise mechanism by which LIF regulates p21 in GC remains unclear, Humbert et al. identified a LIF/STAT3/p21 signaling axis in melanoma, wherein LIF phosphorylates STAT3 to promote p21 transcription, ultimately suppressing tumor growth [49].

The pleiotropic nature of LIF and LIFR signaling presents a significant challenge in defining its precise role in GC. While mounting evidence supports its pro‐tumorigenic function, select studies indicate context‐dependent tumor‐suppressive effects [16]. To resolve these contradictions, future research should focus on quantifying the relative activity of oncogenic versus tumor‐suppressive LIF and LIFR pathways, thereby identifying potential therapeutic contexts in which LIF blockade or activation may be beneficial in GC treatment.

## 7 Future Directions in LIF/LIFR Research for Gastric Cancer

Tumor‐derived LIF was identified as a critical driver of chemoresistance through TAM‐dependent mechanisms. Co‐culture with murine bone marrow–derived macrophages revealed that tumor‐associated macrophages (TAMs) significantly increased the IC50 of cisplatin in AGS gastric cancer cells, more tumoral LIF expression further enhanced TAM‐induced resistance of tumor cells to cisplatin, but the deficiency of LIF in tumor cells obviously diminished this effect of TAMs indicating their role in reducing tumor cell chemosensitivity [35]. Tumor cells overexpressing LIF amplified this TAM‐induced resistance, while LIF deficiency attenuated it. Importantly, neutralizing extracellular LIF with anti‐LIF antibodies restored chemosensitivity, reduced colony formation, and inhibited tumor growth when treated with cisplatin, emphasizing the paracrine function of LIF in chemoresistance. These findings suggest that blocking LIF signaling could enhance the efficacy of chemotherapy in GC. Future research should focus on the molecular mechanisms underlying LIF‐mediated chemoresistance. While the interaction between LIF and TAMs has been established, the specific downstream pathways that drive this effect remain unclear. Investigating the involvement of additional signaling cascades such as PI3K/AKT and Rap1 may reveal novel therapeutic targets that can modulate LIF‐induced resistance [13].

Given its role in activating key oncogenic pathways such as JAK/STAT3, MAPK/ERK, and Hippo/YAP, as well as its ability to promote chemoresistance through TAMs, targeting LIF signaling represents a promising therapeutic strategy [22]. Preclinical studies have demonstrated that EC359 suppresses tumor growth in breast cancer models and patient‐derived xenografts, as well as inhibiting gastric adenocarcinoma progression when used in combination with a TAM inhibitor; expanding on these preclinical studies could further explore the impact of EC359 on GC TMEs, as well as its mechanisms of resistance and predictive biomarkers [25]. Cristina Di Giorgio et al. reported resulted from sequencing 31 patients with gastric cancer to evaluate LIF/LIFR's role in gastric cancer progression. The cell cycle analysis revealed the EC359 in combination with LIF effectively reverses the effect of LIF in a statistically significant manner and decreased the rate of proliferation in gastric cancer cells. Additionally, EC359 was also increased the rate of apoptosis and downregulated E‐cadherin [37]. Additionally, conducting clinical trials may establish its efficacy in combination with standard of care treatments, such as chemotherapy or FGFR4 inhibitors [25].

LIF has been shown to suppress CD8+ T cell infiltration by promoting an immunosuppressive TME through TAMs [38]. In murine models, anti‐LIF therapy significantly increased CD8+ T cell infiltration by 2.4‐fold and reduced TAM populations by 47%, thus reversing immune suppression. Additionally, CXCL9 expression increased 3.5‐fold following LIF blockade, highlighting its role in recruiting cytotoxic T cells. Anti‐PD‐L1 therapies complement this response by restoring exhausted T cell function. Notably, the combination of anti‐LIF and anti‐PD‐L1 therapy led to tumor regression in 80% of treated mice, with a subset exhibiting complete tumor clearance. Moreover, dual blockades more than doubled median overall survival compared to control groups given only a single agent. Future studies should aim to identify patient populations with high LIF expression and optimize combination regimens. An expanded understanding of LIF may enhance immunotherapy efficacy in tumors with poor T cell penetration and improve clinical outcomes.

The LIF/LIFR pathway activates key oncogenic cascades including Hippo–YAP and STAT3 that contribute to EMT, proliferation, and suppression of antitumor immune responses. These effects are particularly pronounced in GC with PC—a subtype associated with poor prognosis and limited response to standard therapies.

While LIF is widely recognized for its tumor‐promoting effects, it also paradoxically influences tumor suppression and apoptosis, underscoring the complexity of its function. Its role in GC remains understudied, and comprehensive molecular profiling to identify tumors with elevated LIF/LIFR signaling is essential for developing precision therapies. Preliminary data suggest that high LIFR expression is associated with worse outcomes in advanced GC, while LIF expression may correlate with improved responsiveness to chemoimmunotherapy and favorable remodeling of the tumor immune microenvironment. These context‐dependent effects make LIF a compelling biomarker for patient stratification and therapeutic targeting.

Importantly, further research is needed to elucidate the mechanisms of action and resistance to LIF/LIFR inhibitors like EC359. Longitudinal studies will help identify compensatory pathways such as FGFR, IL‐6/STAT3, and HIF1α that may limit monotherapy efficacy. Co‐targeting these pathways may enhance therapeutic durability and prevent resistance. Moreover, rational combination strategies, including LIFR inhibition with immune checkpoint blockade (e.g., anti–PD‐1/PD‐L1) or TAM reprogramming agents, may yield synergistic anti‐tumor effects, particularly in “cold” tumors with poor CD8+ T‐cell infiltration.

## 8 Conclusion

LIF and LIFR are critical yet complex regulators of GC progression, influencing oncogenic pathways, immune evasion, and therapeutic resistance. While LIF promotes tumor proliferation, chemoresistance, and immune suppression via JAK/STAT3, MAPK/ERK, and Hippo‐YAP signaling, its context‐dependent tumor‐suppressive roles add layers of complexity. Preclinical studies have highlighted the potential of targeting LIF signaling, either through LIFR inhibition or combination therapies, to restore immune activity and enhance treatment efficacy. Small‐molecule inhibitors alongside immune checkpoint blockades offer promising avenues for further exploration. Future research should focus on identifying predictive biomarkers and clarifying the dual roles of LIF/LIFR to optimize precision therapies. Designing clinical trials to evaluate LIF‐targeted strategies will be essential in translating these insights into improved outcomes for GC patients.

## Author Contributions

**Daniel Park:** writing – original draft, conceptualization, investigation, writing – review and editing. **Kyung‐Il Kim:** conceptualization, investigation, writing – original draft, writing – review and editing. **Yate‐Ching Yuan:** writing – review and editing, conceptualization. **Pranati Shah:** writing – review and editing, conceptualization, visualization. **Hannah Zhong:** writing – original draft, investigation. **Yu‐Han Chen:** writing – review and editing, conceptualization, writing – original draft. **Sharity Ondrejik:** writing – review and editing. **Ellen Choi:** writing – review and editing. **Sofia Guzman:** writing – original draft. **Vitor Goes:** writing – original draft. **Christiana Crook:** formal analysis, writing – original draft, validation, writing – review and editing. **Wenqi Wang:** writing – review and editing, validation. **Dani Ran Castillo:** writing – original draft, project administration, supervision, validation, investigation, formal analysis, writing – review and editing, conceptualization. **Daneng Li:** investigation, project administration, supervision, writing – original draft, conceptualization, validation, writing – review and editing, formal analysis.

## Disclosure

No copyrighted or previously published material requiring permission for reproduction has been used in this review. All figures, tables, and content are original or derived from open‐access sources with proper attribution.

## Ethics Statement

The authors have nothing to report.

## Conflicts of Interest

Daneng Li reports institutional grants or contracts from AstraZeneca and personal consulting fees from AbbVie, AstraZeneca, Bayer, Boehringer Ingelheim, Coherus Biosciences, Eisai, Elevar Therapeutics, Exelixis, Genentech, Jazz Pharmaceuticals, Merck, Merus, Sumitomo Pharma, Transthera Sciences, and Trisalus Life Sciences. All other authors declare no competing interests.

## Data Availability

This study is a review article and does not involve the generation of new data. All data referenced in this work are derived from publicly available published sources, which are properly cited in the references section. No new datasets were generated or analyzed for this manuscript.
